# Gossypiboma versus Gossip-Boma

**DOI:** 10.1155/2011/705062

**Published:** 2011-10-16

**Authors:** Charanjeet Singh, Mamta Gupta

**Affiliations:** Department of Radiology, Zulekha Hospital, P.O. Box no. 48577, Dubai, United Arab Emirates

## Abstract

Gossypiboma, or a retained surgical sponge, is a rare condition, and it can occur after any surgical intervention that requires use of internal swabs. A case of an eight-year-old girl is presented, who had right minithoracotomy for ASD closure. She was finally diagnosed to have a retained surgical sponge in the right pleural cavity.

## 1. Introduction

Gossypiboma, or a retained surgical sponge, is a rare condition which can occur after any surgical intervention that requires use of internal swabs. It is very unusual to forget a surgical sponge in an operated wound, but rarely it is possible despite the extreme cautions of the surgical team and then the consequences can be very severe. The retained surgical sponge can present in any way for example, like a mass, abscess, or clinical picture of bowel obstruction if left intraperitoneally. The most of the cases of Gossypiboma are the patients of postlaparotomy due to any reason, but cases of Gossypiboma of other parts of body for example, thorax, thigh, and neck had also been reported.

## 2. Case Report

We present a case of an eight-year-old girl, a postoperated case of ASD closure. The ASD repair was done through right minithoracotomy in the fourth intercostal space, at mid axillary line. The child was on the routine followup, about two months after the surgery. Mother of the child complained that her daughter was having fever for the last fifteen days, which was not associated with chills or rigors. Clinical examination revealed that the patient was febrile. The basal region of right hemithorax was dull on percussion; on auscultation, decreased air entry and bronchial breathing were present in this region. X-ray chest PA view showed an inhomogeneous radio-opacity in the right lower zone with no definitive signs of volume loss or air broncho-gram. Thin, radio-dense lines were seen in center of the radio-opacity and thought to be the radio-opaque marker of retained surgical sponge. An urgent unenhanced CT scan of thorax was done, which revealed a large extrapulmonary, intrapleural, hypodense space-occupying lesion in the basal part of right hemithorax, having areas of entrapped air bubbles. Thin, coiled structures of high density (average density 440 HU), representing the radio opaque marker of the retained surgical, were noted in core of the lesion. Adjacent lower lobe of right lung was partially collapsed. On reopening the thorax, a retained surgical sponge, surrounded by the fluid and granulation tissue was taken out.

## 3. Discussion

Gossypiboma or cottonoid describes a mass in the body, composed of a cotton matrix, which is surrounded by a foreign body reaction and commonly refers to a retained surgical sponge [1, 2]. Presently synthetic material has replaced the cotton, and the term Textiloma has also been used in literature. The word Gossypiboma is derived from Gossypium (Latine), that is, the cotton and the Boma (Swahili), that is, the place of concealment. We can understand it, in another way also as Gossip-Boma, that is, a mass that may result from gossips of the surgical team during surgery. The oversight of a foreign body during any surgery is rare but can sometimes occur despite the extreme cautions of the surgical team. Most of the cases of Gossypiboma are the patients of post laparotomy due to any reason, but cases of Gossypiboma of other parts of body, for example, thorax, thigh, and neck have also been reported. A retained cotton matrix inside the body produces a local inflammation on the first day, which produces a granulomatous reaction after a week and fibrosis formation after a fortnight [3]. Gossypiboma is a diagnostic dilemma as they may present as asymptomatic to a severe life-threatening condition. Depending upon the site of retention, the signs and symptoms can be an abdominal mass, subacute intestinal obstruction, fistulae, breathlessness, pleural effusion, and consolidation of adjacent lung. A Gossypiboma should be considered in the differential diagnosis of an atypical thick- or thin-walled mass, located in any part of body, in a patient who has undergone a previous surgery. This is probably the most important step in making the diagnosis of a Gossypiboma. Gossypiboma is a rare but important iatrogenic complication of intrathoracic surgery. The pleural space is the most likely site of surgical sponge retention [2, 3]. To the best of our knowledge, only few cases of intra-thoracic Gossypiboma have been described in the literature till date. The swab within the pleural spaces acts as a nidus, and chronic inflammatory changes may develop in the adjacent lung resulting in infolding of lung, which superficially resembles an intrapulmonary abscess or an aspergilloma on CT scan images [3, 4].

**Figure 1:**
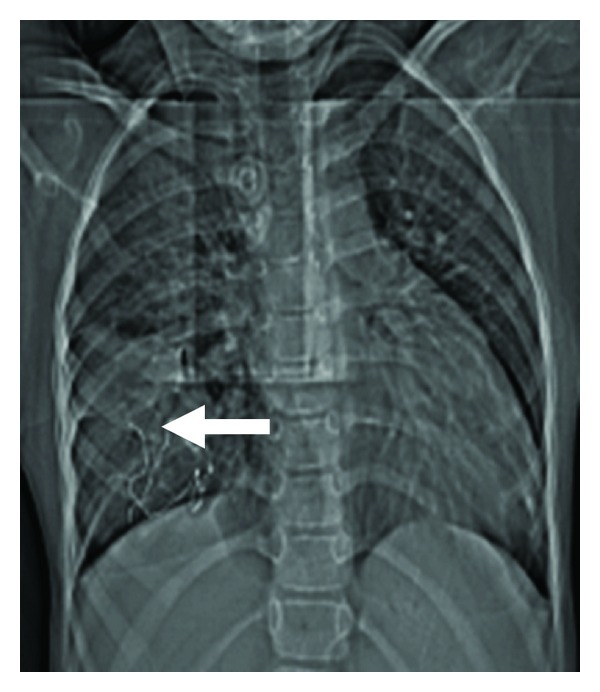
CT scanogram, showing a rounded inhomogeneous radio-opacity in the right lower hemithorax. The center of the lesion is having thin, comparatively dense lines, representing the radio-opaque markers in the retained surgical sponge (Arrow).

**Figure 2:**
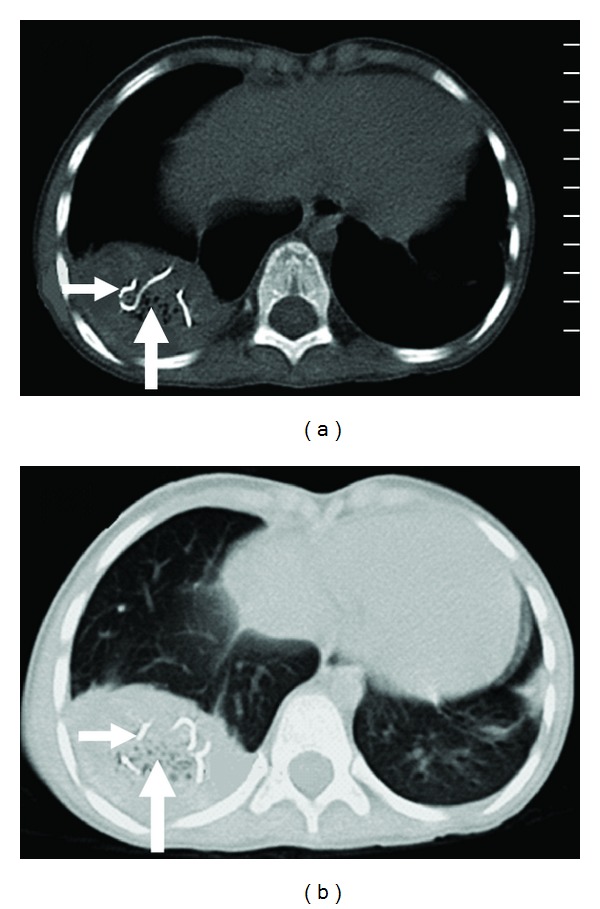
Un-enhanced CT scan section of the thorax, in bone window (a) and lung window setting (b) showing a well-defined, hypodense, pleural based mass in the right lower hemithorax. The whorl-like structure is representing the sponge itself, which is having air droplets (Large Arrows) and a thin, coiled structure of high density in core of the lesion, representing the radio-opaque markers (Small Arrows).

**Figure 3:**
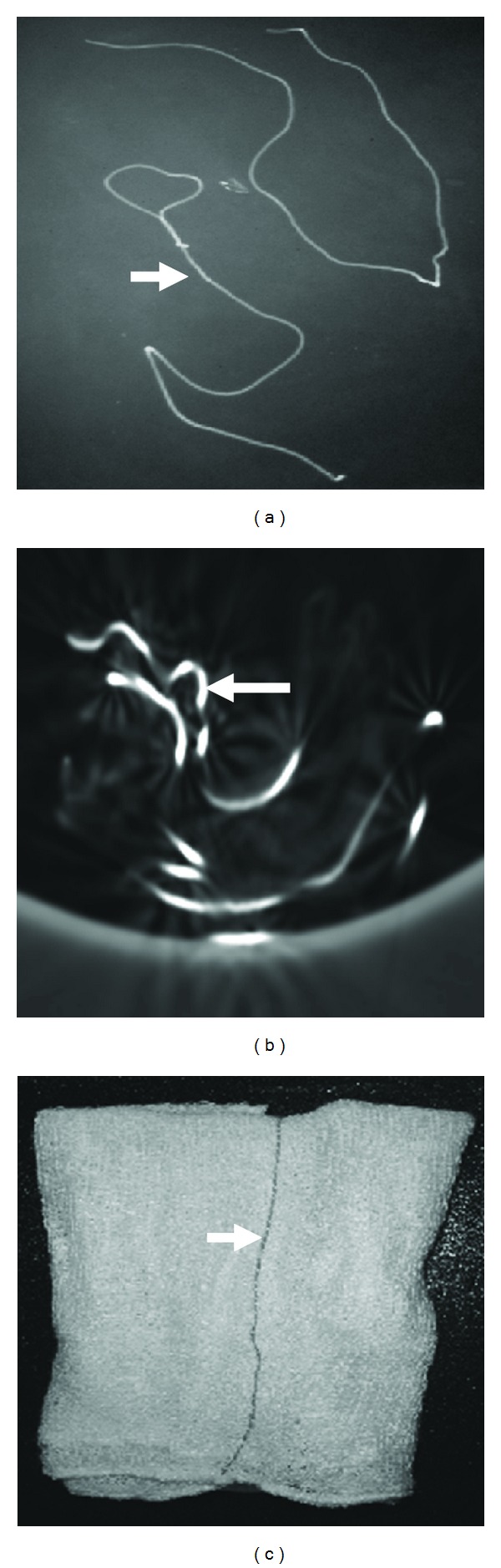
Plain X-ray (a), CT scan (axial section) of the surgical sponge (b), in lung window setting and the photograph of the surgical sponges, having radio-opaque markers (c). Radio-opaque markers are indicated by arrows.

Gossypiboma may have an inconsistent radiological appearance, which is determined by the time in situ, the type of material used, and the anatomic location of the surgical wound. Radiological features of Gossypiboma on plain X-ray include a whorl-like heterogeneous mass, which can be calcified, containing both dense material and air bubbles [3]. As most of the surgical sponges have a radio-opaque marker, if it is not broken in to pieces, plain X-ray is the cheapest and the surest way to reach the correct diagnosis [5] (Figures 1 and 3). Sonography may show some additional help in the diagnosis, but usually is nondiagnostic [3]. CT scan is the method of choice in evaluation of Gossypiboma [2]. The CT scan shows a sharply, well-defined, rounded, low-density mass of inhomogeneous texture, with thick or thin wall, having a dense central part and rim enhancement, which can be indistinguishable from the abscess or a tumor [6]. The typical spongiform pattern with air bubbles is the most characteristic sign on CT images [1, 2, 7] (Figure 2). However, in the early postoperative period, this pattern may mimic the appearance of gel-foam particles placed to control intraoperative haemorrhage or may be confused with a complicated haematoma or an abscess [7–10]. In a chronic, long-standing case, the pattern may mimic the appearance of an echinococcal cyst or an intracavitary fungus ball with formation of loose mycelial fronds [9, 11]. There is no enhancement of the center of the lesion, which is already inhomogeneously dense [1–3, 12]. The high-density center of Gossypiboma is likely to be a trapped clot within the stroma, and the enhancement after injection of iodinated contrast media is due to the inflammatory reaction [13]. Sometimes air bubbles may not be as prominent features of intrathoracic Gossypiboma, as they are in intraabdominal Gossypiboma. This may be due to resorption of air by the pleura [14]. These characteristic appearances of the Gossypiboma may be variable with any imaging technique and the diagnosis can be especially difficult if the radio-opaque marker is not present [15] if the marker is disturbed by folding, twisting, or disintegration over a period [13], or if the marker is misinterpreted as a calcification or a surgical suture [14]. MRI appearance of Gossypiboma has been reported recently using T2W images [14, 16]. The findings of transthoracic core biopsy may be helpful by showing the characteristic cotton fibers [14].

The special feature of our case was the radio-opaque marker of surgical sponge, seen as thin coiled, radio-opaque shadows on chest X-ray (Figure 1). The findings were also reinforced by CT scan of thorax, marker seen lying in core of the Gossypiboma (Figure 2). In situ radiological appearance of radio-opaque marker of the retained surgical sponge has been reported only in few cases so far [17]. The presence of the typical air bubbles also made our way easy to diagnose it as a case of Gossypiboma. The findings were confirmed on re-opening the thorax. 

In summary, we can say that intrathoracic Gossypiboma is a rare iatrogenic complication that can have severe medical consequences [3, 18–20]. Radio-opaque marker of retained surgical sponge if visualized is enough to conclude the diagnosis even on plain X-ray. Sometimes the retained intra thoracic sponges do not have the characteristic radiological appearance; it may not be easy to diagnose them, even in a patient with a history of surgery, and radiologist may find it difficult to make a preoperative diagnosis. The actual numbers of reported cases of Gossypiboma are definitely significantly below the true incidences of these cases, and the same is true for their complications because of the legal implications [21]. This paper can be seen as a reminder to our surgical teams to be very careful during any surgery. They should never forget to do proper swab and instrument count before closure of any surgical wound so that they must be fully ensured that their patient is not going to get a GOSSIP-BOMA.
